# Genetic Diversity, Recombination, and Pathogenicity of Porcine Epidemic Diarrhea Virus Strains Circulating in China During 2023–2024

**DOI:** 10.1155/tbed/1340053

**Published:** 2026-05-19

**Authors:** Yunlei Cao, Ahui Cui, Xin Chen, Yang Cheng, Rongfeng Tang, Hui Zeng, Yuchen Li, Qian Yang

**Affiliations:** ^1^ MOE Joint International Research Laboratory of Animal Health and Food Safety, College of Veterinary Medicine, Nanjing Agricultural University, Weigang 1, Nanjing, 210095, Jiangsu, China, njau.edu.cn

**Keywords:** genetic recombination, molecular epidemiology, pathogenicity, phylogenetic analysis, porcine epidemic diarrhea virus

## Abstract

Porcine epidemic diarrhea virus (PEDV) is an enteric alphacoronavirus that causes severe diarrhea and high mortality in neonatal piglets, posing a persistent threat to the global swine industry. Despite extensive vaccination, PEDV continues to circulate in China, underscoring the need for updated molecular epidemiological surveillance. Here, we investigated the prevalence, genetic diversity, and pathogenicity of PEDV strains circulating in China during 2023–2024. A total of 714 clinical samples were collected from diarrheic pigs in seven major swine‐producing regions. PEDV RNA was detected in 36.27% of samples, with substantial positivity observed in both anal and nasal swabs. Phylogenetic analysis based on the full‐length S gene revealed that circulating strains clustered into multiple subgroups, including GIa, GIIa, GIIb, and GIIc, with GIIc being predominant, indicating pronounced genetic diversity and co‐circulation of distinct lineages. Two PEDV strains were isolated from PEDV‐positive intestinal tissues and designated PEDV‐HeiHo‐2024 (GIIb) and PEDV‐NJZU‐2024 (GIa). Recombination analysis identified PEDV‐HeiHo‐2024 as a putative recombinant derived from distinct GII lineage strains. Comparative analyses of key neutralizing epitopes and N‐linked glycosylation sites in the S protein revealed substantial divergence from classical vaccine strains. Experimental infection of neonatal piglets demonstrated that PEDV‐HeiHo‐2024 induced severe diarrhea and characteristic intestinal lesions, confirming its high pathogenicity, whereas PEDV‐NJZU‐2024 showed no apparent pathogenic effects. Collectively, these findings highlight the continued circulation of genetically diverse and pathogenic PEDV strains in China and emphasize the importance of sustained molecular surveillance and vaccine updates.

## 1. Introduction

Porcine epidemic diarrhea virus (PEDV), a highly contagious enteric alphacoronavirus, causes severe watery diarrhea and dehydration in neonatal piglets, with mortality rates reaching up to 100%, thereby inflicting continuous and significant economic losses on the swine industry [1–3]. Since the emergence and rapid dissemination of highly pathogenic variants in China in 2010, PEDV has caused recurrent epidemics worldwide. Despite the widespread implementation of vaccination programs, effective control of these outbreaks remains a challenge [4–6]. Previous studies have indicated that variations in the PEDV S gene—particularly insertions, deletions, and amino acid substitutions—are closely associated with the virus’s transmissibility, immune evasion capabilities, and pathogenicity [7–9]. Therefore, continuous surveillance of the genetic characteristics of currently circulating PEDV strains, coupled with the evaluation of changes in their pathogenicity, is critical for understanding viral evolutionary trends and optimizing prevention and control strategies.

As a member of the genus alphacoronavirus within the family Coronaviridae, PEDV possesses a single‐stranded, positive‐sense RNA genome approximately 28 kb in length [10]. The viral genome encodes multiple non‐structural and structural proteins. Among these, the S protein plays a pivotal role in viral attachment, receptor binding, and membrane fusion, while also serving as the primary antigenic determinant responsible for eliciting neutralizing antibody responses in the host [11–13]. Substantial research indicates that genetic variations in the S gene—including point mutations, insertions, and deletions—are closely correlated with alterations in PEDV antigenicity, shifts in tissue tropism, and changes in virulence [14–16]. Consequently, systematic phylogenetic and molecular characterization based on the S gene has become a crucial approach for elucidating the evolutionary dynamics of circulating PEDV strains and assessing the potential risks of vaccine failure.

Due to the lack of proofreading activity of the coronavirus RNA‐dependent RNA polymerase (RdRp), PEDV continuously accumulates genetic mutations during replication, leading to the formation of highly diverse viral populations [17]. Based on phylogenetic analysis of the S gene, circulating PEDV strains are typically categorized into two major genogroups: classical (GI) and variant (GII). These are further delineated into multiple subgroups, including GIa, GIb, GIIa, GIIb, and GIIc [18–20]. In recent years, numerous surveillance studies have demonstrated a complex co‐circulation pattern of distinct PEDV subgroups worldwide, with the GII lineage remaining predominant [21–23]. The coexistence of multiple PEDV lineages (especially GIIa, GIIb, and vaccine‐associated strains) in the same geographic area and time period poses a significant challenge to disease control, as it can complicate diagnosis, vaccine selection, and the emergence of novel recombinant strains. Notably, the detection rate of the GIIc subgroup—a lineage associated with S‐INDEL recombination events—has shown an upward trend in certain regions, indicating that PEDV is undergoing continuous genetic remodeling [24, 25]. However, systematic characterization of the genetic features and pathogenic changes of recently circulating PEDV strains, particularly through comprehensive analyses based on viral isolates, remains relatively limited.

To characterize the recent molecular epidemiology and evolutionary dynamics of PEDV, we conducted systematic surveillance of diarrheic clinical samples collected from seven major swine‐producing regions in China, including Heilongjiang, Shanxi, Fujian, Guangdong, Sichuan, Liaoning, and Jiangsu provinces. Two PEDV strains were successfully isolated from PEDV‐positive intestinal tissues and were classified into distinct genetic subgroups, designated PEDV‐HeiHo‐2024 (GIIb) and PEDV‐NJZU‐2024 (GIa). We further focused on the genetic characterization of PEDV‐HeiHo‐2024, defining its phylogenetic relationship with representative reference strains and assessing its pathogenicity using a neonatal piglet infection model. Together, these analyses reveal substantial genetic diversity and ongoing evolution among recently circulating PEDV strains in China and provide experimental evidence for understanding their molecular epidemiological features and pathogenic differences.

## 2. Results

### 2.1. Detection and Molecular Characterization of PEDV in Diarrheic Pigs

To generate S gene sequence data for subsequent analyses, total RNA was extracted from PEDV‐positive clinical samples and used for amplification of the full‐length S gene. Given the length of the S gene, its coding region was covered by four overlapping amplicons generated using specific primer sets (Supporting Information 1: Table S1). As shown in Supporting Information 2: Figure S1, agarose gel electrophoresis revealed single bands of the expected sizes for all four fragments, while no amplification was observed in negative controls, indicating specific and reliable recovery of S gene fragments suitable for sequencing.

Between 2023 and 2024, a total of 714 clinical samples were collected from diarrheic pigs in seven provinces of China, including Heilongjiang, Shanxi, Fujian, Guangdong, Sichuan, Liaoning, and Jiangsu. PEDV RNA was detected in 259 samples, corresponding to an overall positivity rate of 36.27% (Supporting Information 3: Table S2).

**Figure 1:**
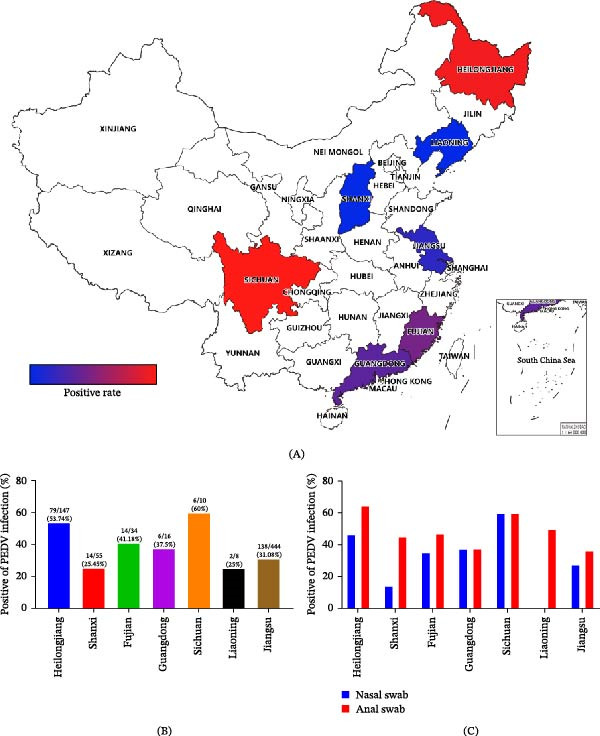
Geographic distribution of PEDV detection in diarrheic pigs. (A) Geographic distribution of PEDV positivity rates across seven provinces in China from 2023 to 2024. Color intensity from blue to red indicates increasing PEDV positivity rates. (B) Provincial PEDV positivity rates based on all tested clinical samples. Numbers above bars indicate the number of PEDV‐positive samples relative to the total number tested for each province. (C) PEDV positivity rates in nasal swabs (blue) and anal swabs (red) collected from diarrheic pigs in each province.

PEDV detection rates varied across provinces (Figure 1A). At the provincial level, positivity rates exceeded 50% in Heilongjiang (53.74%) and Sichuan (60.0%), whereas lower detection rates were observed in Shanxi (25.45%) and Liaoning (25.0%). Intermediate positivity rates were detected in Fujian (41.18%), Guangdong (37.5%), and Jiangsu (31.08%) (Figure 1B).

When samples were stratified by specimen type, PEDV RNA was consistently detected in both nasal and anal swabs across all sampled provinces (Figure 1C). Overall, the positivity rate was 30.97% (131/423) in nasal swabs and 43.99% (128/291) in anal swabs.

### 2.2. Phylogenetic Classification of PEDV S Gene Sequences

From the 259 PEDV‐positive clinical samples, 22 full‐length S gene sequences were successfully amplified and sequenced. These sequences were analyzed together with 50 representative reference strains using maximum‐likelihood phylogenetic analysis based on the full‐length S gene.

**Figure 2:**
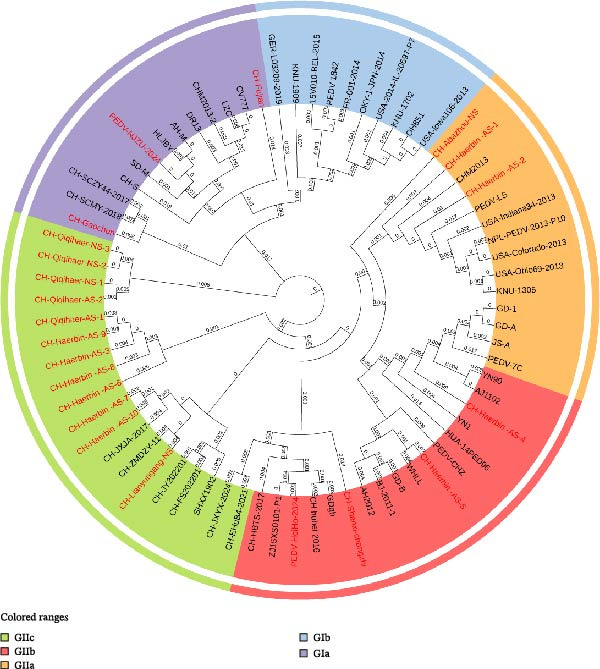
Phylogenetic analysis of PEDV S gene sequences. A maximum‐likelihood phylogenetic tree was constructed based on full‐length PEDV S gene sequences using MEGA version 12 with 1000 bootstrap replicates. The 22 full‐length S gene sequences obtained in this study are highlighted in red. Colored outer rings indicate different PEDV genogroups and subgroups.

The resulting phylogenetic tree showed that the PEDV sequences obtained in this study clustered into four established subgroups, including GIa, GIIa, GIIb, and GIIc, consistent with previously reported classification schemes (Figure 2). Specifically, three sequences clustered within the GIa subgroup, three within GIIa, four within GIIb, and the majority of sequences (*n* = 12) grouped within the GIIc subgroup.

### 2.3. Isolation and Identification of PEDV Strains

**Figure 3:**
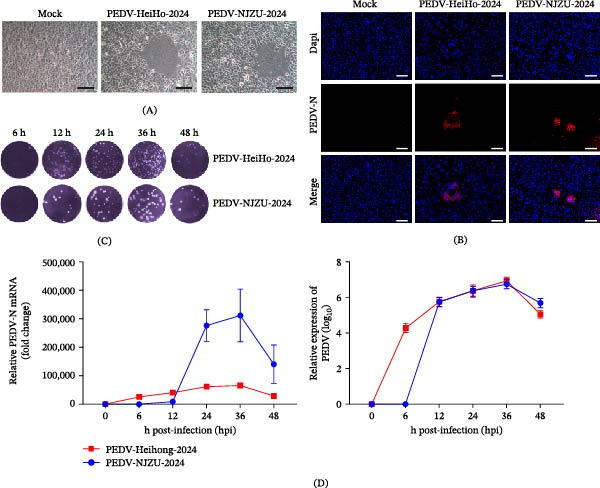
Isolation and in vitro characterization of PEDV‐HeiHo‐2024 and PEDV‐NJZU‐2024. (A) CPE observed in Vero cells infected with PEDV‐HeiHo‐2024 or PEDV‐NJZU‐2024 compared with mock‐infected cells. Scale bars, 20 μm. (B) Immunofluorescence analysis of PEDV‐infected Vero cells. PEDV N protein is shown in red, and nuclei are counterstained with DAPI (blue). Scale bars, 20 μm. (C) Plaque assays showing infectious virus production in culture supernatants at the indicated time points post infection. (D) Replication kinetics of PEDV‐HeiHo‐2024 and PEDV‐NJZU‐2024 in Vero cells, as determined by quantitative RT‐PCR analysis of PEDV N gene RNA levels and by quantification of infectious virus titers at the indicated time points.

PEDV‐positive intestinal samples were subjected to virus isolation in Vero cells. After serial passage, infected cells developed characteristic cytopathic effects (CPE), including cell rounding, fusion, and syncytium formation, whereas no cytopathic changes were observed in mock‐infected controls (Figure 3A).

Successful isolation of PEDV was further confirmed by RT‐PCR detection of viral RNA in culture supernatants. Two PEDV isolates were obtained and designated PEDV‐HeiHo‐2024 and PEDV‐NJZU‐2024. Immunofluorescence analysis demonstrated specific staining of the PEDV nucleocapsid (N) protein in Vero cells infected with either isolate, while no specific fluorescence signal was detected in mock‐infected cells (Figure 3B).

Growth kinetics analysis showed that both PEDV‐HeiHo‐2024 and PEDV‐NJZU‐2024 replicated efficiently in Vero cells (Figure 3C). Infectious virus titers increased over time and reached peak levels at 36 h post infection, with maximal titers of approximately 1 × 10^6.3^ plaque‐forming unit (PFU)/mL (Figure 3D).

### 2.4. Nucleotide and Amino Acid Sequence Identity of PEDV Isolates

To assess the genetic relatedness of the two PEDV isolates, nucleotide and deduced amino acid sequences of the S gene from PEDV‐HeiHo‐2024 and PEDV‐NJZU‐2024 were compared with those of representative PEDV reference strains retrieved from GenBank.

PEDV‐HeiHo‐2024 shared high nucleotide sequence identity in the S gene with reference strains, ranging from 96.5% to 99.1%, and exhibited the highest similarity to the GIIb strain AH2012 (Supporting Information 4: Figure. S2A). Consistently, amino acid sequence comparisons showed high identity between PEDV‐HeiHo‐2024 and GIIb lineage strains, including AH2012 (98.9%), AJ1102 (98.5%), GDS28 (99.1%), and MN (98.9%) (Supporting Information 4: Figure S2B).

In contrast, PEDV‐NJZU‐2024 displayed S gene nucleotide identities ranging from 94.8% to 99.9% when compared with reference strains and showed the greatest similarity to the classical GIa strain SD‐M (Supporting Information 4: Figure S2A). Amino acid sequence analysis further demonstrated high identity between PEDV‐NJZU‐2024 and GIa strains, including SD‐M (99.9%) and CV777 (97.9%) (Supporting Information 4: Figure S2B).

### 2.5. Comparison of Antigenic Epitopes and N‐Linked Glycosylation Sites in the PEDV S Protein

To compare antigenic features of the newly isolated PEDV strains with those of reference viruses, amino acid sequences of the S protein from PEDV‐HeiHo‐2024 and PEDV‐NJZU‐2024 were aligned with those of the classical vaccine strain CV777 and the GII reference strain AJ1102. Previously reported antigenic epitopes, including S10, COE, SS2, SS6, and 2C10, were examined for sequence variation (Supporting Information 5: Figure S3).

Sequence comparison revealed distinct substitution patterns within antigenic regions of the S protein (Supporting Information 6: Figure S4A,B). Relative to CV777, PEDV‐NJZU‐2024 contained eight amino acid substitutions within the S10 region. Within the COE region, seven amino acid differences were identified when compared with the conserved motifs of the vaccine strain (Supporting Information 6: Figure S4C,D). In addition, two amino acid substitutions were observed in the SS6 region, whereas no substitutions were detected in the SS2 or 2C10 epitopes of PEDV‐NJZU‐2024.

In contrast, comparison between PEDV‐HeiHo‐2024 and AJ1102 showed four amino acid substitutions within the S10 region. Three amino acid differences were identified within the COE region, located at residues Ile^499^, Ser^520^, and Ile^560^ (Supporting Information 6: Figure S4E,F). No amino acid substitutions were detected in the SS2, SS6, or 2C10 epitope regions of PEDV‐HeiHo‐2024.

**Figure 4:**
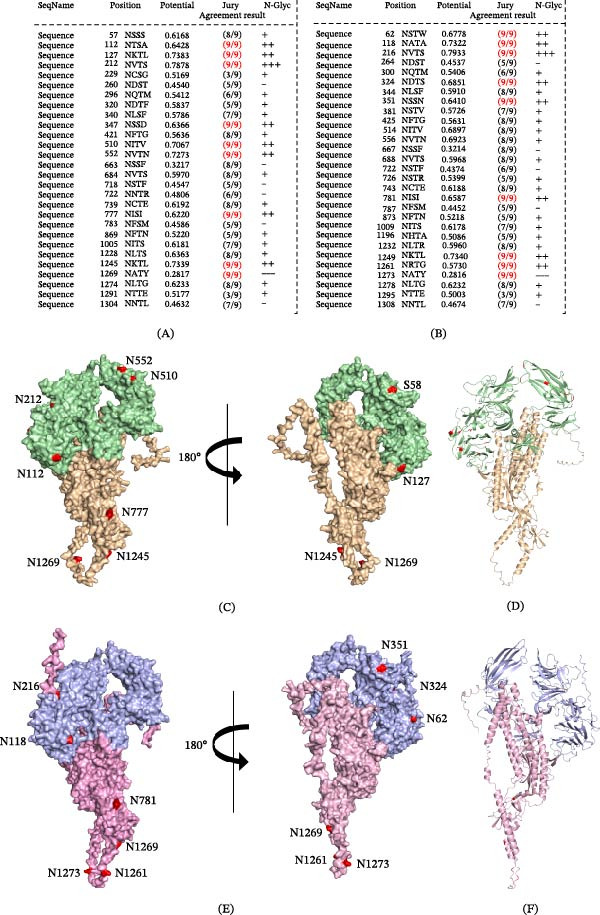
Structural mapping of predicted N‐linked glycosylation sites in the PEDV S protein. Schematic summaries of predicted N‐linked glycosylation sites in the S proteins of PEDV‐NJZU‐2024 and PEDV‐HeiHo‐2024, respectively (A, B). Atomic surface and cartoon representations of the S protein monomers of PEDV‐NJZU‐2024 (C, D) and PEDV‐HeiHo‐2024 (E, F), with predicted N‐linked glycosylation sites highlighted in red.

Predicted N‐linked glycosylation sites within the S protein were further analyzed using the NetNGlyc server (Figure 4A,B). Compared with CV777, PEDV‐NJZU‐2024 contained an additional predicted N‐linked glycosylation site at position 112 within the N‐terminal domain (motif NTSA), while predicted glycosylation sites at positions 321 (NDTS) and 1258 (NRTG) were absent (Figure 4C,D). In comparison with AJ1102, PEDV‐HeiHo‐2024 lacked a predicted N‐linked glycosylation site at position 514 (motif NITV) (Figure 4E,F).

### 2.6. Recombination Analysis of PEDV‐HeiHo‐2024

**Figure 5:**
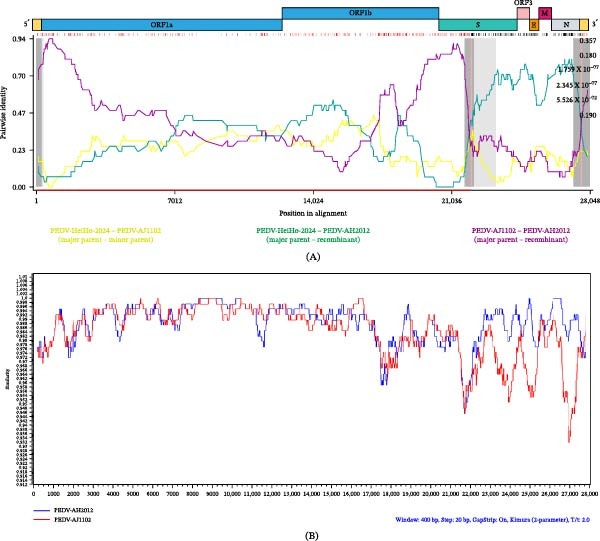
Recombination analysis of PEDV‐HeiHo‐2024. (A) Similarity plot generated by RDP4 showing pairwise sequence identity across the PEDV genome. The analysis was supported by at least six independent detection methods. The green and yellow lines represent reference strains AH2012 (KC210145) and AJ1102 (JX188454), respectively. (B) SimPlot analysis illustrating recombination breakpoints across the genome of PEDV‐HeiHo‐2024.

Potential recombination events in PEDV‐HeiHo‐2024 were examined using RDP4 and SimPlot analyses. RDP4 detected a significant recombination signal in the genome of PEDV‐HeiHo‐2024, supported by at least six independent detection methods (Figure 5A). The query strain was highly sequence similar to the non‐recombinant genome region, and phylogenetic clustering of the query strain was performed using breakpoint‐defined fragments. Representative reference strains from the major prevalent lineages (GIIb and vaccine‐associated groups) identified in our study were also included. The analysis identified AH2012 as the major parental strain and AJ1102 as the minor parental strain.

The putative recombination breakpoints were mapped to a genomic region spanning the S gene and extending toward the N gene, corresponding to nucleotide positions 22,026–27,597. This recombination pattern was further supported by SimPlot analysis, which showed alternating regions of higher sequence similarity between PEDV‐HeiHo‐2024 and the two parental reference strains across the breakpoint region (Figure 5B).

### 2.7. Pathogenicity of Two PEDV Isolates in Neonatal Piglets

The pathogenicity of the two PEDV isolates was evaluated using a neonatal piglet infection experiments. Piglets inoculated with PEDV‐HeiHo‐2024 developed clinical manifestations characteristic of porcine epidemic diarrhea (PED) within 24 h post‐inoculation, including watery diarrhea, vomiting, and lethargy, whereas mock‐infected piglets remained clinically normal throughout the observation period.

**Figure 6:**
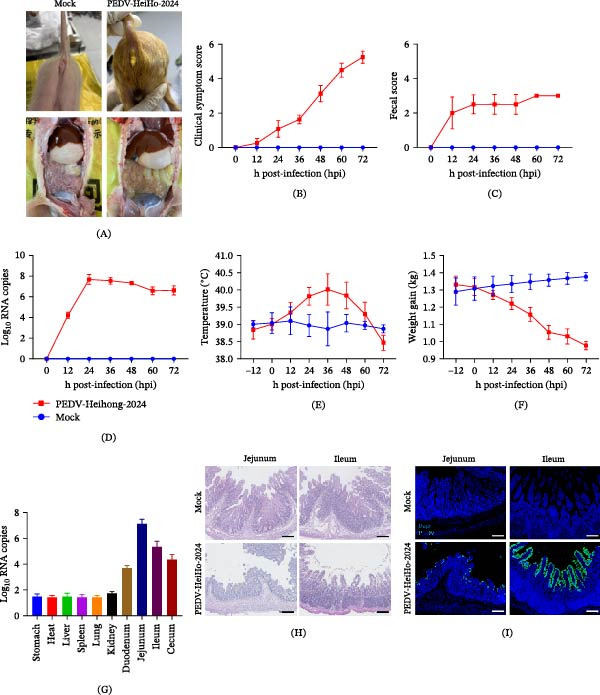
Pathogenicity of PEDV‐HeiHo‐2024 in neonatal piglets. (A) Representative images showing clinical appearance and gross intestinal lesions in mock‐infected and PEDV‐HeiHo‐2024–infected piglets. (B) Clinical symptom scores of piglets following infection (score 0, healthy; scores 1–2, mild; scores 3–4, moderate; scores 5–6, severe). (C) Diarrhea scores of piglets after infection (score 0, normal; score 1, mild; score 2, moderate; score 3, severe). (D) Fecal shedding of PEDV RNA determined by quantitative RT‐PCR. (E) Rectal temperature changes in piglets during the course of infection. (F) Body weight changes of piglets following infection. (G) Viral RNA loads in different tissues of PEDV‐HeiHo‐2024–infected piglets, as measured by quantitative RT‐PCR. (H) Histopathological changes in the jejunum and ileum after PEDV challenge, assessed by H&E staining. Intestinal tissues from mock‐infected piglets display normal villus architecture, whereas tissues from PEDV‐HeiHo‐2024–infected piglets show villus atrophy. Scale bars, 20 μm. (I) Immunofluorescence detection of PEDV N protein in the jejunum and ileum. PEDV N protein is shown in green, and nuclei are counterstained with DAPI (blue). No specific signal is detected in mock‐infected tissues, whereas positive staining is observed in intestinal epithelial cells of PEDV‐HeiHo‐2024–infected piglets. Scale bars, 20 μm.

Gross pathological examination of PEDV‐HeiHo‐2024–infected piglets revealed typical intestinal lesions, including gastric distension with accumulated gas and fluid, thinning of the intestinal wall, and watery intestinal contents (Figure 6A). PEDV‐HeiHo‐2024 infection significantly increased clinical symptom and diarrhea scores in piglets (Figure 6B,C). Quantitative RT‐PCR analysis of fecal samples revealed persistent PEDV RNA shedding in infected piglets, while no specific viral RNA signal was detected in the mock group (Figure 6D). Rectal temperature fluctuated during infection (Figure 6E), furthermore, weight gain was affected in infected piglets, showing marked weight loss (Figure 6F). To further clarify the tissue distribution of the virus, quantitative RT‐PCR was performed on different tissues. The results showed that PEDV RNA was detectable in various tissues of infected piglets, with particularly high viral load in intestinal‐associated tissues, especially the small intestine (Figure 6G). Histopathological analysis further demonstrated marked viral enteritis in the small intestine, particularly in the jejunum and ileum, characterized by villus shortening, atrophy, and partial villus loss (Figure 6H). Consistent with these findings, immunofluorescence analysis detected PEDV antigen in villous epithelial cells of the jejunum and ileum in PEDV‐HeiHo‐2024–infected piglets, whereas no specific fluorescence signal was observed in tissues from mock‐infected piglets (Figure 6I).

In contrast, piglets inoculated with PEDV‐NJZU‐2024 did not develop diarrhea or other clinical signs during the observation period. No apparent gross or histological lesions were observed in intestinal tissues, and immunofluorescence analysis did not detect PEDV antigen in the jejunum or ileum of PEDV‐NJZU‐2024–infected piglets (Supporting Information 7: Figure S5).

## 3. Discussion

PED remains a major cause of economic loss in the global swine industry [26]. Following the emergence of highly pathogenic PEDV strains in China in 2010, the virus has continued to play a prominent role in outbreaks of viral diarrhea in pigs [27]. Although vaccination has been widely implemented, accumulating epidemiological evidence indicates that PEDV has not been eliminated from swine populations and continues to circulate under current production systems, highlighting the persistent challenges associated with its control [28, 29]. In this study, we conducted systematic surveillance of diarrheic pig samples collected from seven regions of China during 2023–2024. The detection rate of PEDV in these samples was comparable to that reported in previous studies, indicating ongoing viral circulation in contemporary swine herds. Furthermore, two PEDV isolates, PEDV‐HeiHo‐2024 and PEDV‐NJZU‐2024, were successfully recovered and subsequently characterized. By integrating molecular analyses with neonatal piglet infection experiments, this study provides a combined genetic and phenotypic assessment of recently circulating PEDV strains.

PEDV RNA was detected in nasal swab samples from a subset of diarrheic piglets, indicating that, in addition to intestinal specimens, PEDV can also be detected in samples associated with the upper respiratory tract. The presence of PEDV RNA in nasal swabs may result from multiple nonexclusive routes. Under field conditions, PEDV is abundantly shed in feces, and contaminated fecal material or bedding can generate virus‐containing dust or aerosols that may be inhaled and transiently detected in the nasal mucosa [30]. Alternatively, following primary oral infection, local mucosal spread may contribute to the temporary presence of viral RNA in the nasal cavity [31]. Although PEDV is classically regarded as a fecal–orally transmitted virus, our previous experimental studies demonstrated that PEDV can enter the host via the nasal route under controlled conditions and subsequently establish intestinal infection [32], providing biological plausibility for the nasal detection observed here. The detection of PEDV RNA in nasal swabs may indicate an upper respiratory tract infection or a transient presence of the virus. However, RNA detection alone cannot prove that the virus is actively replicating in nasal tissue. Importantly, detection of PEDV RNA in nasal swabs should be interpreted as an epidemiologically informative observation rather than direct evidence of respiratory transmission. From a control perspective, these results suggest that PEDV surveillance should consider potential nasal exposure and environmental factors alongside traditional fecal–oral transmission routes.

The S protein is a key determinant of PEDV entry into host cells and of neutralizing antibody recognition, and sequence variation in this protein is closely linked to viral pathogenicity and epidemiological behavior [33]. Accordingly, the S gene is commonly used to assess the genetic diversity and evolutionary dynamics of PEDV. Phylogenetic analysis of S gene sequences from 22 PEDV‐positive samples together with 50 reference strains revealed that contemporary PEDV strains circulating in China are distributed among multiple subgroups, including GIa, GIIa, GIIb, and GIIc, indicating substantial genetic diversity and lineage coexistence. Consistent with previous reports describing the predominance of GIIc strains in recent Chinese outbreaks, most sequences in this study clustered within the GIIc lineage. The coexistence of multiple subtypes in the same space‐time provides a substrate for recombination events. In addition, the isolate PEDV‐HeiHo‐2024 was assigned to the GIIb subgroup, suggesting continued diversification and active circulation within the GII lineage. Notably, several sequences, including the isolate PEDV‐NJZU‐2024, clustered within the classical GIa subgroup. Similar persistence of GIa strains under conditions dominated by GII variants has been reported in northeastern China, indicating that GIa strains have not been completely displaced but may continue to co‐circulate with variant lineages in the current immune landscape [34]. PEDV‐NJZU‐2024 has extremely high sequence homology (99.9%) with the classic SD‐M strain and may represent a vaccine‐associated SD‐M‐like virus. Further evidence (e.g., confirmed vaccination history, epidemiological links, broader surveillance, or other genetic markers associated with attenuation) is needed to support the conclusion that it is a naturally occurring low‐virulence wild‐type strain. At the molecular level, both PEDV‐HeiHo‐2024 and PEDV‐NJZU‐2024 exhibited distinct features in key antigenic epitopes and N‐linked glycosylation sites of the S protein when compared with the classical vaccine strain CV777 and the variant reference strain AJ1102, reflecting ongoing antigenic diversification among circulating PEDV strains. Previous studies have shown that amino acid substitutions within the S1 subunit, particularly in the COE region, may alter antigenic conformation and antibody interactions [35]. The substitutions observed in the COE region of the isolates in this study therefore provide a molecular context for potential antigenic differences. In addition, alterations in N‐linked glycosylation sites may influence epitope exposure through steric effects [36], thereby affecting host immune recognition, a phenomenon also observed among the isolates analyzed here [22]. These observations suggest that the evolution of PEDV in China does not follow a simple lineage replacement pattern. Rather, multiple lineages appear to persist and evolve in parallel, underscoring the need for continued molecular surveillance to monitor ongoing viral diversification and potential inter‐lineage recombination.

In this study, two PEDV isolates, PEDV‐HeiHo‐2024 and PEDV‐NJZU‐2024, were successfully recovered and characterized. Whole‐genome and S protein sequence analyses further indicated that PEDV‐HeiHo‐2024 is genetically closely related to representative highly pathogenic GII strains such as AH2012 and AJ1102 [37, 38]. Recombination analysis identified a recombination signal in the genome of PEDV‐HeiHo‐2024, with putative breakpoints located within the S gene–associated region (nt 22,026–27,597). Similar recombination‐driven diversification phenomena have been confirmed in multiple studies: the S protein D0 domain has been identified as a high‐frequency recombination hotspot, and recombination in this region may affect immune escape by regulating the virus’s ability to bind sialic acid. Recombination events occurring in the S gene have been reported to generate chimeric S proteins with altered antigenic properties [39], When the recombination breakpoint is located in the S gene‐related region, it is more likely to affect antigenicity and virulence phenotype. S1 contains receptor binding and major neutralizing epitopes. If recombination replaces regions such as COE, SS2, and SS6, or alters N glycosylation sites, it may reduce cross‐neutralization, leading to decreased immune protection. Alternatively, it may increase cross‐reactivity due to the fragment being closer to the vaccine strain, depending on parental origin and epitope differences. S2 participates in membrane fusion. If recombination affects fusion efficiency and stability, it can alter the intestinal epithelial replication rate and tissue damage, thereby affecting the severity of diarrhea. In the context of ongoing PEDV circulation, detection of such recombination events suggests continued genetic reshaping among GII lineages and underscores the importance of sustained molecular surveillance to identify and monitor emerging recombinant variants. Although both isolates replicated efficiently in Vero cells and induced typical CPE, their pathogenicity in neonatal piglets differed markedly. Under the current epidemiological background, PEDV isolates circulating in the field can exhibit substantial heterogeneity in pathogenicity. Previous studies have shown that, compared with the highly pathogenic GII strains that have dominated recent global outbreaks, some GIa strains tend to display reduced pathogenicity in piglet models [40, 41]. The difference in pathogenicity between the two viruses may be due to changes at multiple levels, including the S protein. PEDV‐NJZU‐2024 has a seven amino acid substitution in its COE region, with an additional predicted N‐glycosylation site at position 112 of the N‐terminus, which may affect epitope exposure through steric hindrance. PEDV‐HeiHo‐2024, on the other hand, lacks a glycosylation site at position 514, and more importantly, its S gene region contains recombination signals. Recombination can produce chimeric S proteins, altering receptor binding or membrane fusion efficiency. Previous studies have confirmed that the S protein D0 domain is a recombination hotspot, and its variations can regulate intestinal colonization and pathogenicity [25], mutations at glycosylation sites are sufficient to reduce piglet mortality from 100% to 33% [16]. S gene recombination combined with glycosylation changes likely contributes to the distinctly different pathogenic phenotypes of the two viruses. Further research is needed to utilize reverse genetics to interchange recombinant fragments or individual glycosylation sites between the two viruses, thereby accurately verifying the independent contribution of these genetic differences to pathogenicity.

In summary, surveillance of diarrheic pig samples collected from seven regions of China during 2023–2024 indicates that PEDV remains widely detected in the field. S gene–based phylogenetic analysis highlights substantial genetic heterogeneity within the contemporary PEDV population, with multiple subgroups, including GIa, GIIa, GIIb, and GIIc, co‐circulating during the same epidemic period, while the GII lineage continues to predominate. Within this context, two PEDV isolates representing distinct subgroups were successfully recovered, among which the GIIb isolate PEDV‐HeiHo‐2024 exhibited pronounced pathogenicity in neonatal piglets. However, only two infectious strains were successfully isolated in cell culture, and the limited number of isolates may not fully reflect the genetic and phenotypic diversity of the PEDV epidemic population. Further sequence and recombination analyses showed that this isolate harbors recombination events and associated variation within the S gene region, suggesting that antigen‐related regions of circulating PEDV strains remain subject to ongoing genetic change. Lineage co‐circulation provides genetic material for recombination, which drives the continuous diversification of viruses by modifying the S protein functional domain, and ultimately manifests as a significant differentiation in pathogenicity. From a disease control perspective, such variability underscores the need for continued molecular surveillance to monitor the emergence of strains with altered biological properties. Overall, by integrating field surveillance with phylogenetic characterization and functional evaluation of representative isolates, this study offers a refined view of the genetic diversity and evolutionary behavior of PEDV currently circulating in China.

## 4. Materials and Methods

### 4.1. Cells, Viruses, and Antibodies

Vero cells were maintained in Dulbecco’s modified Eagle’s medium (DMEM; Biochannel, China) supplemented with 10% fetal bovine serum (FBS; Biochannel, China) and 1% penicillin–streptomycin at 37°C in a humidified incubator with 5% CO_2_. The wild‐type PEDV strain Zhejiang08, previously identified as clustering with emerging virulent strains, was preserved in our laboratory.

A monoclonal antibody against the PEDV N protein was custom generated by GenScript (Nanjing, China) and used at a dilution of 1:200 for immunofluorescence assays. Alexa Fluor 594–conjugated donkey anti‐mouse IgG1 (1:200; 34112ES60, Yeasen, China) and Alexa Fluor 488–conjugated goat anti‐mouse IgG1 (1:200; 33206ES60, Yeasen, China) were used as secondary antibodies. For nuclear counterstaining, 4^′^, 6‐diamidino‐2‐phenylindole (DAPI; 1:1000; Thermo Fisher Scientific, USA) was applied. Slides were mounted using an antifade mounting medium (Yeasen, China). Unless otherwise specified, all other reagents were purchased from Sigma–Aldrich (St. Louis, MO, USA).

### 4.2. Clinical Sample Collection

From 2023 to 2024, a total of 714 clinical samples were collected from pigs exhibiting diarrheic symptoms in seven major swine‐producing regions of China, including Heilongjiang, Shanxi, Fujian, Guangdong, Sichuan, Liaoning, and Jiangsu provinces. The samples consisted of 423 nasal swabs and 291 anal swabs and were collected for routine diagnostic purposes.

Nasal and rectal swabs were collected from piglets with clinical diarrhea using sterile rayon swabs. For nasal swabs, the swab was inserted deeply into the nasal cavity and rotated gently against the mucosa. For rectal swabs, the swab was inserted into the rectum and rotated to ensure the collection of visible fecal material. Immediately after collection, each swab was immersed in a sterile tube containing 1.5 mL of phosphate‐buffered saline (PBS) (pH 7.2). The samples were transported to the laboratory at 4°C within 24 h. To obtain the supernatant, the tubes containing the swabs were vortexed vigorously for 1 min to suspend the viral particles. The suspension was then centrifuged at 5000 × *g* for 10 min at 4°C to remove gross debris. The clarified supernatant was collected and transferred to fresh 1.5 mL microcentrifuge tubes. The processed samples were stored at −80°C until RNA extraction.

### 4.3. Viral RNA Extraction

Total RNA was extracted from samples using TRIzol reagent (Vazyme, China) according to the manufacturer’s instructions. Reverse transcription was performed using HiScript IV RT SuperMix for qPCR (+gDNA wiper; Vazyme, China) following a two‐step protocol. Briefly, 1 µg of total RNA was mixed with 3 µL of 5 × gDNA wiper Mix and RNase‐free water to a final volume of 15 µL and incubated at 42°C for 2 min to remove residual genomic DNA. Subsequently, 5 µL of 4 × HiScript IV qRT SuperMix was added, and reverse transcription was carried out at 37°C for 15 min, followed by enzyme inactivation at 85°C for 5 s.

### 4.4. Detection of PEDV‐Positive Samples and S Gene Sequencing

Total RNA was extracted from clinical samples or cell culture supernatants using TRIzol reagent (Vazyme, China). Reverse transcription was performed using the HiScript IV RT SuperMix for qPCR with gDNA wiper (Vazyme, China) according to the manufacturer’s instructions. PEDV RNA was detected by RT‐PCR using primers specific for the PEDV N gene (Supporting Information 1: Table S1).

PEDV‐positive samples were subsequently used for amplification of the full‐length S gene. To facilitate sequencing, the S gene was amplified as four overlapping fragments (designated S1, S2, S3, and S4) using specific primers listed in Supporting Information 1: Table S1. PCR products were analyzed by electrophoresis on 1% agarose gels, and bands of the expected size were excised and purified using a gel extraction kit (Omega Bio‐Tek, USA). Purified PCR products were subjected to Sanger sequencing by Sangon Biotech (Shanghai, China).

### 4.5. Geographic Analysis of PEDV Infection

The geographic distribution of PEDV infection was analyzed based on the sampling locations of PEDV‐positive clinical specimens. PEDV positivity rates were calculated for each sampled province and visualized to illustrate regional differences in virus detection. Data visualization was performed using GraphPad Prism version 10.0, Adobe Illustrator 2022, and the ggplot2 package in R. Geographic base maps were obtained from DataV (accessed on June 11, 2025).

### 4.6. Phylogenetic and Recombination Analyses

Phylogenetic relationships of PEDV strains were inferred based on the full‐length S gene sequences using MEGA X version 12.0. Maximum likelihood (ML) phylogenetic trees were constructed under the best‐fit nucleotide substitution model, with branch support assessed by 1000 bootstrap replicates. Alignment positions with less than 95% site coverage, including gaps, missing data, or ambiguous bases, were excluded from the analysis. Reference PEDV sequences used in this study are listed in Supporting Information 8: Table S3.

Potential recombination events were screened using the RDP4 software package by applying multiple detection methods, including RDP, GENECONV, BootScan, MaxChi, Chimaera, SiScan, and 3Seq. Recombination signals supported by at least six methods were considered credible. Putative recombination events were further validated and visualized using SimPlot to generate similarity plots.

### 4.7. Sequence and Structural Analysis of the PEDV S Protein

Amino acid sequences of the PEDV S protein were aligned using MegAlign to compare previously reported major neutralizing epitopes, including S10, COE, SS2, SS6, and 2C10, among field isolates and reference strains. Amino acid substitutions within these epitope regions were analyzed to assess potential antigenic variation.

Potential N‐linked glycosylation sites in the S protein were predicted using the NetNGlyc 1.0 server. Only sites with prediction scores greater than 0.5 and supported by all nine neural network predictions were considered.

For structural analysis, S protein amino acid sequences were retrieved from the NCBI database and submitted to the AlphaFold server for structure prediction. The top‐ranked model (Model 0) was selected for subsequent analysis and visualization using PyMOL version 2.6.

### 4.8. Virus Isolation and Titration

Vero cells were seeded into 12‐well plates and cultured to 80%–90% confluence. Clarified intestinal tissue homogenates were mixed at a 1:1 ratio with DMEM containing 10 μg/mL trypsin (Sigma–Aldrich, USA), and 0.5 mL of the mixture was inoculated onto the cell monolayers. After adsorption at 37°C for 1 h, the inoculum was removed, cells were washed twice with serum‐free DMEM, and maintenance medium containing 5 μg/mL trypsin was added. Cells were incubated at 37°C with 5% CO_2_ and monitored daily for CPE. Virus stocks were blind‐passaged for up to five passages until stable CPE was observed. Viral replication was confirmed by RT‐PCR detection of the PEDV N gene in culture supernatants.

Viral titers were determined by plaque assay. Vero cells were seeded into 12‐well plates to form confluent monolayers and inoculated with 10‐fold serial dilutions of virus‐containing supernatants. After adsorption at 37°C for 1 h, the inoculum was removed and cells were overlaid with DMEM containing 2% FBS and 0.7% agarose. Plates were incubated at 37°C with 5% CO_2_ for 3 days, followed by crystal violet staining to visualize and count viral plaques.

### 4.9. Histopathology and Immunofluorescence Analysis

Intestinal tissue samples were fixed in 4% paraformaldehyde for 24 h, dehydrated through a graded ethanol series, embedded in paraffin, and sectioned at a thickness of 5 μm. Sections were stained with hematoxylin and eosin (H&E) for histopathological examination.

For immunofluorescence analysis, paraffin sections were deparaffinized and rehydrated, followed by antigen retrieval in citrate buffer (pH 6.0) at 90–95°C for 15 min. Sections were permeabilized with 0.1% Triton X‐100, blocked with 5% FBS, and incubated overnight at 4°C with a mouse monoclonal antibody against the PEDV N protein. After washing, sections were incubated with Alexa Fluor 488–conjugated secondary antibodies and counterstained with DAPI. Fluorescence signals were visualized using a fluorescence microscope.

### 4.10. Animal Challenge Experiments

Duroc × Landrace × Yorkshire crossbred piglets were obtained from a commercial swine farm in Jiangsu Province, China. Prior to experimental infection, all piglets were confirmed to be seronegative for PEDV, porcine reproductive and respiratory syndrome virus (PRRSV), porcine deltacoronavirus (PDCoV), and transmissible gastroenteritis virus (TGEV).

For infection studies, 3‐day‐old piglets were randomly assigned into three groups (*n* = 4 per group): mock‐infected control, PEDV‐HeiHo‐2024‐infected, and PEDV‐NJZU‐2024‐infected. Piglets in the PEDV challenge groups were orally inoculated with 1 × 10^6^ PFUs of PEDV. Clinical signs, including diarrhea and overall sickness, were scored daily using a standardized scoring system (diarrhea score: 0–3, 0: normal feces, 1: soft but formed feces, 2: semi‐liquid feces, 3: watery diarrhea [42, 43]; clinical symptom score: 0–6, 0: healthy, 1–2: mild [lethargy and reduced activity], 3–4: moderate [inactivity and mild dehydration], 5–6: severe [severe dehydration, hunched posture, and inability to stand]) [44, 45]. At 72 h post‐infection (hpi), piglets were humanely euthanized, and tissue samples were collected from the jejunum and ileum for subsequent analyses.

## 5. Ethical Statement

All animal experiments were approved by the Institutional Animal Care and Use Committee of Nanjing Agricultural University (approval number: NJAULLSC2024093) and were conducted in accordance with the National Institutes of Health guidelines for the care and use of laboratory animals.

## 6. Statistical Analysis

All data are presented as mean ± standard deviation (SD). Statistical analyses were performed using SPSS software (version 17.0; SPSS Inc., USA). For comparisons among multiple groups, one‐way analysis of variance (ANOVA) was used, followed by Fisher’s least significant difference (LSD) post hoc test when the overall ANOVA indicated statistical significance. Comparisons between two groups were performed using an unpaired two‐tailed Student’s *t* test. Unless otherwise stated, all experiments were performed with at least three independent biological replicates.

## Author Contributions

Conception of the work: Qian Yang and Yuchen Li. Cellular and animal experiments: Yunlei Cao, Ahui Cui, Xin Chen, Yang Chen, Rongfeng Tang, and Hui Zeng. Data analysis and interpretation, manuscript preparation: Yuchen Li and Yunlei Cao.

## Funding

This work was financially supported by the National Natural Science Foundation of China (Grant 32473063), the Natural Science Foundation of Jiangsu Province (Grant BK20240198), the Fundamental Research Funds for the Central Universities (Grants KJYQ2024008 and KYT2023004), the China Post‐doctoral Science Foundation Funded Project (Grant 2023M731731), and the Priority Academic Program Development of Jiangsu Higher Education Institutions (PAPD), Jiangsu Province Agricultural Science and Technology Independent Innovation Fund Project (Grant CX(24)1010).

## Disclosure

All authors have read and approved the final manuscript.

## Conflicts of Interest

The authors declare no conflicts of interest.

## Supporting Information

Additional supporting information can be found online in the Supporting Information section.

## Supporting information


**Supporting Information 1** Table S1. Sequences of primers used in this study.

**Supporting Information 2** Figure S1. Amplification of the PEDV S gene. The full‐length PEDV S gene was amplified as four overlapping fragments (S1–S4) and analyzed by agarose gel electrophoresis. Lane M, DNA size marker; lane 1, negative control; lane 2, laboratory‐preserved PEDV strain Zhejiang08; and lane 3, PEDV‐positive clinical sample. All four fragments produced single bands of the expected sizes, and no nonspecific amplification was detected in the negative control.

**Supporting Information 3** Table S2. Detection of PEDV in clinical samples collected from diarrheic pigs in China during 2023–2024.

**Supporting Information 4** Figure S2. Nucleotide sequence identity analysis of PEDV isolates. Heat maps showing pairwise nucleotide sequence identities of (A) the S gene and (B) the complete genome between PEDV‐HeiHo‐2024, PEDV‐NJZU‐2024, and representative PEDV reference strains. Percent nucleotide identities are indicated within each cell.

**Supporting Information 5** Figure S3. Amino acid alignment of major antigenic epitopes in the PEDV S protein. Sequence alignments of the S10, COE, SS2, SS6, and 2C10 epitopes are shown for PEDV‐HeiHo‐2024, PEDV‐NJZU‐2024, and representative PEDV reference strains. Dots indicate residues identical to the reference sequence, and letters indicate amino acid substitutions. PEDV‐HeiHo‐2024 and PEDV‐NJZU‐2024 are highlighted in red.

**Supporting Information 6** Figure S4. Structural mapping of amino acid substitutions in the S protein of PEDV isolates. (A) Superimposition of S protein monomer structures of PEDV‐NJZU‐2024 (light green) and the classical strain CV777 (blue). (B) Superimposition of S protein monomer structures of PEDV‐HeiHo‐2024 (light purple) and the GII reference strain AJ1102 (green). (C, D) Visualization of amino acid substitutions within the COE region of the PEDV‐NJZU‐2024 S protein relative to CV777. (E, F) Visualization of amino acid substitutions within the COE region of the PEDV‐HeiHo‐2024 S protein relative to AJ1102. The same color scheme is applied consistently for each strain across all panels.

**Supporting Information 7** Figure S5. Pathogenicity assessment of PEDV‐NJZU‐2024 in neonatal piglets. (A) Histopathological examination of the jejunum and ileum from mock‐infected and PEDV‐NJZU‐2024–infected piglets by H&E staining, showing preserved villus architecture in both groups. Scale bars, 20 μm. (B) Immunofluorescence analysis of jejunum and ileum sections for detection of PEDV N protein. PEDV N protein is shown in green, and nuclei are counterstained with DAPI (blue). No PEDV antigen was detected in intestinal tissues from mock‐infected or PEDV‐NJZU‐2024–infected piglets. Scale bars, 20 μm.

**Supporting Information 8** Table S3. The information about the PEDV reference sequences.

## Data Availability

The authors confirm that all data supporting the findings of this work are available within the study and its Supporting Information section and are fully accessible without restriction.
