# Correction to “Genetic Diversity, Recombination, and Pathogenicity of Porcine Epidemic Diarrhea Virus Strains Circulating in China During 2023–2024”

**DOI:** 10.1155/tbed/9764125

**Published:** 2026-07-14

Y. Cao, A. Cui, X. Chen, et al., “Genetic Diversity, Recombination, and Pathogenicity of Porcine Epidemic Diarrhea Virus Strains Circulating in China During 2023–2024,” *Transboundary and Emerging Diseases*, 2026, 1340053, https://doi.org/10.1155/tbed/1340053.

In the article, there are errors in the keys for the graphs shown in Figures 3D and 6B–F, in which “PEDV‐HeiHo‐2024” was incorrectly written as “PEDV‐Heihong‐2024.” The correct Figures 3 and 6 are shown below:

**Figure 3:**
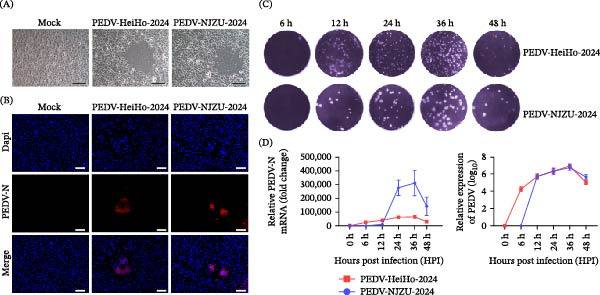
Isolation and in vitro characterization of PEDV‐HeiHo‐2024 and PEDV‐NJZU‐2024. (A) CPE observed in Vero cells infected with PEDV‐HeiHo‐2024 or PEDV‐NJZU‐2024 compared with mock‐infected cells. Scale bars, 20 μm. (B) Immunofluorescence analysis of PEDV‐infected Vero cells. PEDV N protein is shown in red, and nuclei are counterstained with DAPI (blue). Scale bars, 20 μm. (C) Plaque assays showing infectious virus production in culture supernatants at the indicated time points post infection. (D) Replication kinetics of PEDV‐HeiHo‐2024 and PEDV‐NJZU‐2024 in Vero cells, as determined by quantitative RT‐PCR analysis of PEDV N gene RNA levels and by quantification of infectious virus titers at the indicated time points.

**Figure 6:**
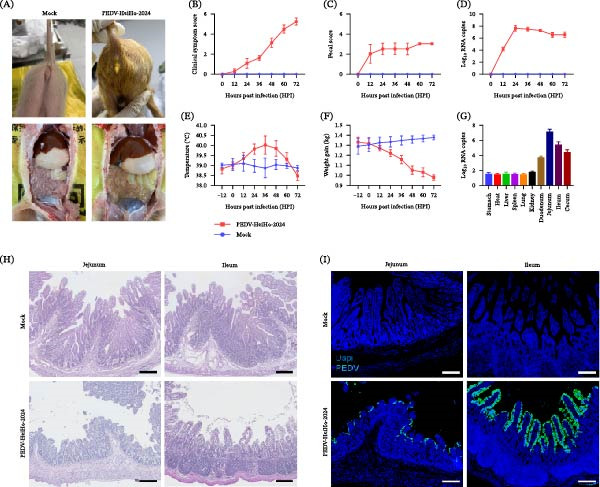
Pathogenicity of PEDV‐HeiHo‐2024 in neonatal piglets. (A) Representative images showing clinical appearance and gross intestinal lesions in mock‐infected and PEDV‐HeiHo‐2024–infected piglets. (B) Clinical symptom scores of piglets following infection (score 0, healthy; scores 1–2, mild; scores 3–4, moderate; scores 5–6, severe). (C) Diarrhea scores of piglets after infection (score 0, normal; score 1, mild; score 2, moderate; score 3, severe). (D) Fecal shedding of PEDV RNA determined by quantitative RT‐PCR. (E) Rectal temperature changes in piglets during the course of infection. (F) Body weight changes of piglets following infection. (G) Viral RNA loads in different tissues of PEDV‐HeiHo‐2024–infected piglets, as measured by quantitative RT‐PCR. (H) Histopathological changes in the jejunum and ileum after PEDV challenge, assessed by H&E staining. Intestinal tissues from mock‐infected piglets display normal villus architecture, whereas tissues from PEDV‐HeiHo‐2024–infected piglets show villus atrophy. Scale bars, 20 μm. (I) Immunofluorescence detection of PEDV N protein in the jejunum and ileum. PEDV N protein is shown in green, and nuclei are counterstained with DAPI (blue). No specific signal is detected in mock‐infected tissues, whereas positive staining is observed in intestinal epithelial cells of PEDV‐HeiHo‐2024–infected piglets. Scale bars, 20 μm.

We apologize for these errors.

