# Supplementary material for: Genetic Diversity, Recombination, and Pathogenicity of Porcine Epidemic Diarrhea Virus Strains Circulating in China During 2023–2024
Source: Transbound Emerg Dis. 2026 May 19;2026:1340053. doi: 10.1155/tbed/1340053 (PMC13184637; doi:10.1155/tbed/1340053)
Supplement: Supplementary file 1 — Supporting Information 1 Table S1. Sequences of primers used in this study. [file TBED-2026-1340053-s007.docx]

Table S1. Sequences of primers used in this study.

| **Primer** | **Sequence** |
| --- | --- |
| JY-PEDV-F | TTCGGTTCTATTCCCGTTGATG |
| JY-PEDV-R | CCCATGAAGCACTTTCTCACTATC |
| JY-TGEV-F | TTACAAACTCGCTATCGCATGG |
| JY-TGEV-R | TGTCACATCACCTTTACCTGC |
| JY-PoRV-F | CCCCGGTATTGAATATACCACAGT |
| JY-PoRV-R | TTTCTGTTGGCCACCCTTTAGT |
| JY-PDCoV-F | ACCAACCAACACCGTCCTTTA |
| JY-PDCoV-R | AGAACCACGAGACTGTAAGCA |
| PEDV-S1-F | ATGACGCCTTTAATTTACTTCTGG |
| PEDV-S1-R | ACAGTAGGAGGTAAAACAGCCA |
| PEDV-S2-F | CTAAGATTTATGGACTAGGCC |
| PEDV-S2-R | GTAGAAGAAACCAGGCAAC |
| PEDV-S3-F | GTTGATTACTGGCACGCCT |
| PEDV-S3-R | GATTGCGATTTGACGCACTC |
| PEDV-S4-F | AACTTCAGCCTTTGAGAGTGT |
| PEDV-S4-R | AAAGACAAGTTGGCAGACTT |
| PEDV-N-F | CACCTCCTGCTTCACGTACA |
| PEDV-N-R | AGCTCCACGACCCTGGTTAT |
| GAPDH-F | TCATCATCTCTGCCCCTTCT |
| GAPDH-R | GTCATGAGTCCCTCCACGAT |
