# Supplementary figures and images for: Genetic Diversity, Recombination, and Pathogenicity of Porcine Epidemic Diarrhea Virus Strains Circulating in China During 2023–2024
Source: Transbound Emerg Dis. 2026 May 19;2026:1340053. doi: 10.1155/tbed/1340053 (PMC13184637; doi:10.1155/tbed/1340053)

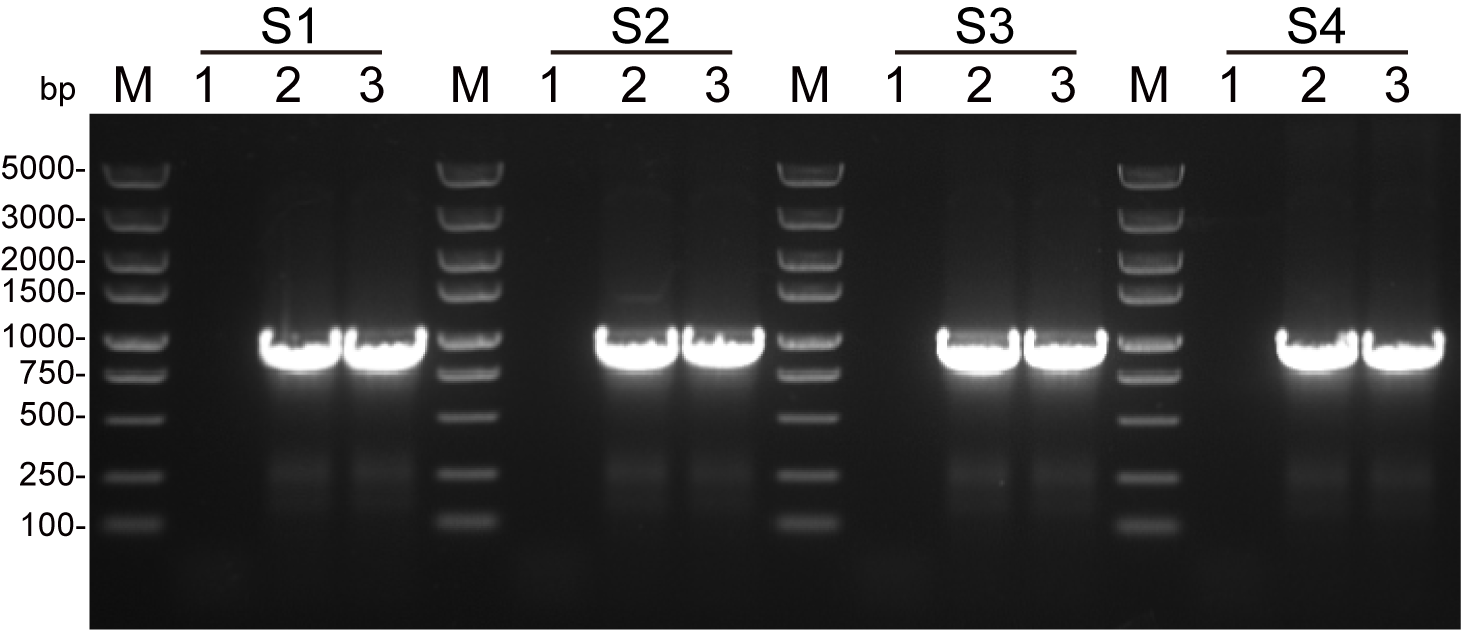

Supplement: Supplementary file 2 — Supporting Information 2 Figure S1. Amplification of the PEDV S gene. The full‐length PEDV S gene was amplified as four overlapping fragments (S1–S4) and analyzed by agarose gel electrophoresis. Lane M, DNA size marker; lane 1, negative control; lane 2, laboratory‐preserved PEDV strain Zhejiang08; and lane 3, PEDV‐positive clinical sample. All four fragments produced single bands of the expected sizes, and no nonspecific amplification was detected in the negative control. [file TBED-2026-1340053-s001.tif]

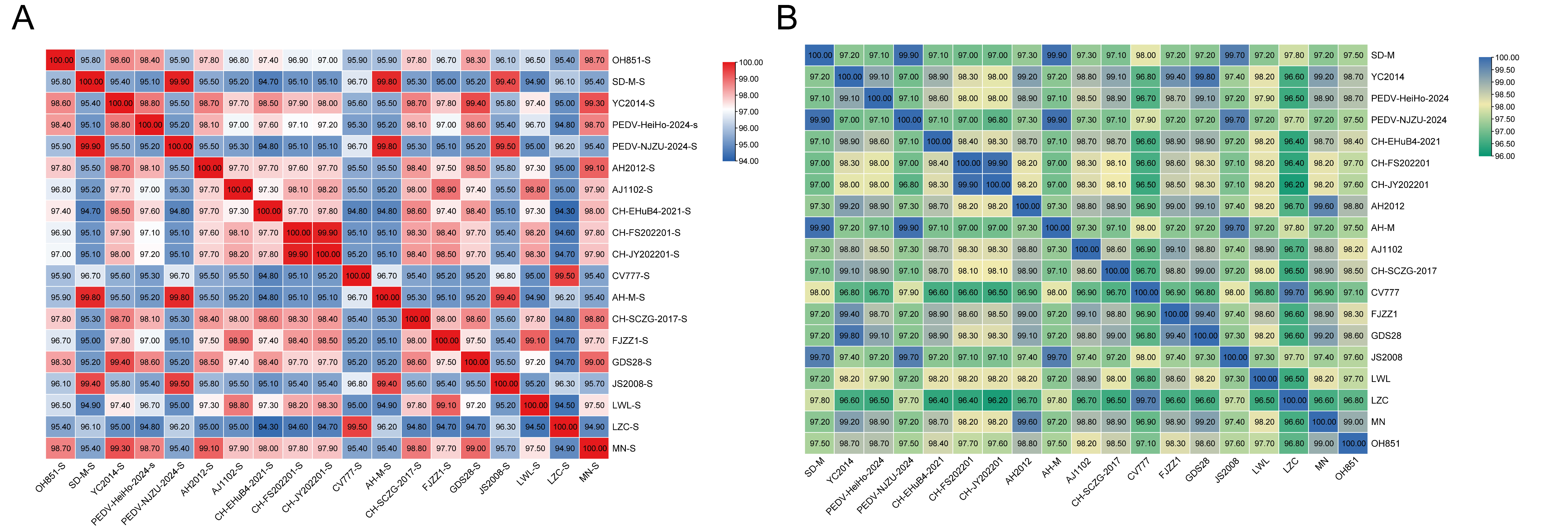

Supplement: Supplementary file 4 — Supporting Information 4 Figure S2. Nucleotide sequence identity analysis of PEDV isolates. Heat maps showing pairwise nucleotide sequence identities of (A) the S gene and (B) the complete genome between PEDV‐HeiHo‐2024, PEDV‐NJZU‐2024, and representative PEDV reference strains. Percent nucleotide identities are indicated within each cell. [file TBED-2026-1340053-s008.tif]

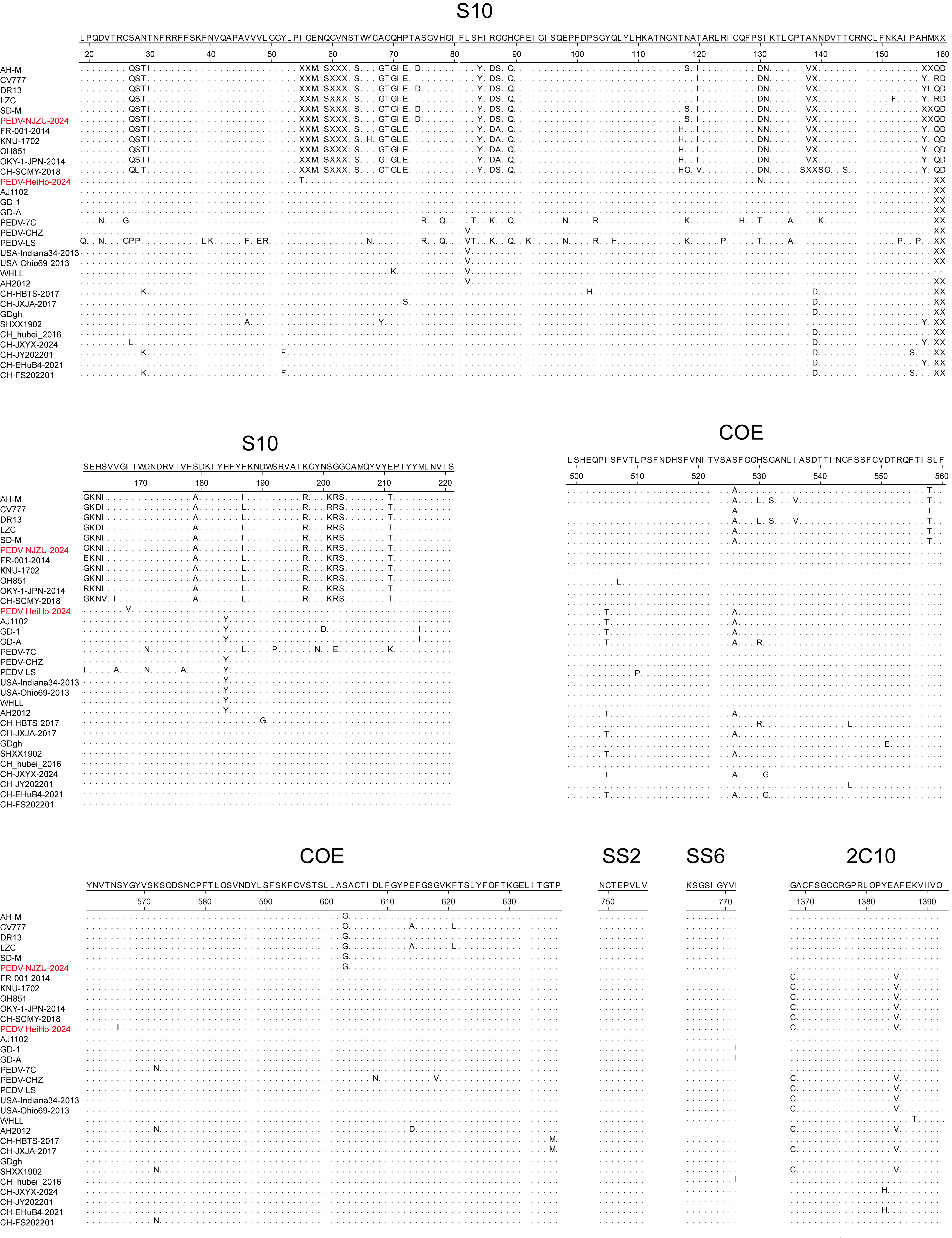

Supplement: Supplementary file 5 — Supporting Information 5 Figure S3. Amino acid alignment of major antigenic epitopes in the PEDV S protein. Sequence alignments of the S10, COE, SS2, SS6, and 2C10 epitopes are shown for PEDV‐HeiHo‐2024, PEDV‐NJZU‐2024, and representative PEDV reference strains. Dots indicate residues identical to the reference sequence, and letters indicate amino acid substitutions. PEDV‐HeiHo‐2024 and PEDV‐NJZU‐2024 are highlighted in red. [file TBED-2026-1340053-s004.tif]

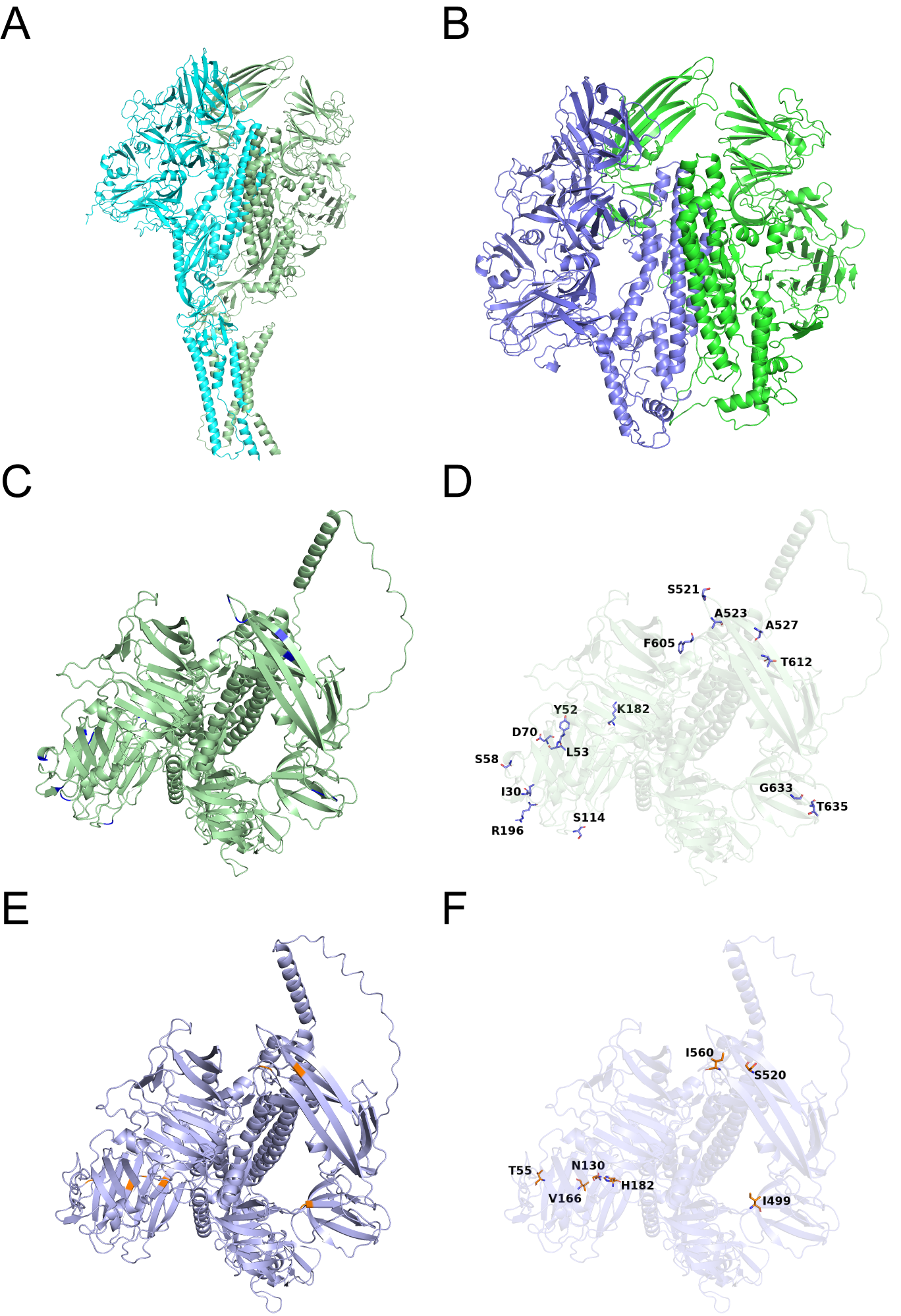

Supplement: Supplementary file 6 — Supporting Information 6 Figure S4. Structural mapping of amino acid substitutions in the S protein of PEDV isolates. (A) Superimposition of S protein monomer structures of PEDV‐NJZU‐2024 (light green) and the classical strain CV777 (blue). (B) Superimposition of S protein monomer structures of PEDV‐HeiHo‐2024 (light purple) and the GII reference strain AJ1102 (green). (C, D) Visualization of amino acid substitutions within the COE region of the PEDV‐NJZU‐2024 S protein relative to CV777. (E, F) Visualization of amino acid substitutions within the COE region of the PEDV‐HeiHo‐2024 S protein relative to AJ1102. The same color scheme is applied consistently for each strain across all panels. [file TBED-2026-1340053-s005.tif]

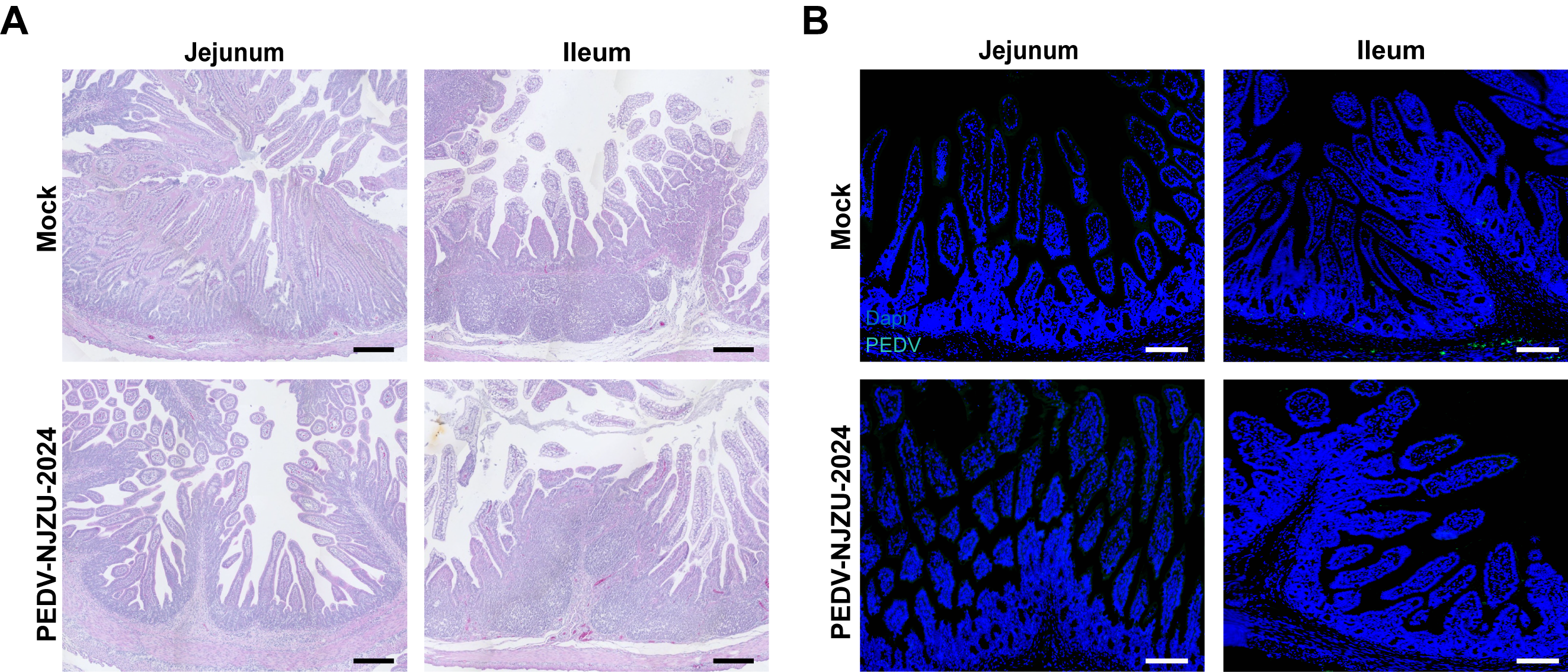

Supplement: Supplementary file 7 — Supporting Information 7 Figure S5. Pathogenicity assessment of PEDV‐NJZU‐2024 in neonatal piglets. (A) Histopathological examination of the jejunum and ileum from mock‐infected and PEDV‐NJZU‐2024–infected piglets by H&E staining, showing preserved villus architecture in both groups. Scale bars, 20 μm. (B) Immunofluorescence analysis of jejunum and ileum sections for detection of PEDV N protein. PEDV N protein is shown in green, and nuclei are counterstained with DAPI (blue). No PEDV antigen was detected in intestinal tissues from mock‐infected or PEDV‐NJZU‐2024–infected piglets. Scale bars, 20 μm. [file TBED-2026-1340053-s006.tif]
