# Supplementary material for: Genetic Diversity, Recombination, and Pathogenicity of Porcine Epidemic Diarrhea Virus Strains Circulating in China During 2023–2024
Source: Transbound Emerg Dis. 2026 May 19;2026:1340053. doi: 10.1155/tbed/1340053 (PMC13184637; doi:10.1155/tbed/1340053)
Supplement: Supplementary file 3 — Supporting Information 3 Table S2. Detection of PEDV in clinical samples collected from diarrheic pigs in China during 2023–2024. [file TBED-2026-1340053-s002.docx]

Table S2. Detection of PEDV in clinical samples collected from diarrheic pigs in China during 2023-2024

| **Total** | **SampleType** | | **Total** | **PEDV Positive Rate**  **Positive/Sample (%)** |
| --- | --- | --- | --- | --- |
|  | **Nasal swab** | **Anal swab** |  |  |
| Heilongjiang | 88 | 59 | 147 | 79/147(53.74%) |
| Shanxi | 35 | 20 | 55 | 14/55(25.45%) |
| Fujian | 17 | 17 | 34 | 14/34(41.18%) |
| Guangdong | 8 | 8 | 16 | 6/16(37.5%) |
| Sichuan | 5 | 5 | 10 | 6/10(60%) |
| Liaoning | 4 | 4 | 8 | 2/8(25%) |
| Jiangsu | 266 | 178 | 444 | 138/444(31.08%) |
| Total | 423 | 291 | 714 | 259/714(36.27%) |
