# Supplementary material for: Genetic Diversity, Recombination, and Pathogenicity of Porcine Epidemic Diarrhea Virus Strains Circulating in China During 2023–2024
Source: Transbound Emerg Dis. 2026 May 19;2026:1340053. doi: 10.1155/tbed/1340053 (PMC13184637; doi:10.1155/tbed/1340053)
Supplement: Supplementary file 8 — Supporting Information 8 Table S3. The information about the PEDV reference sequences. [file TBED-2026-1340053-s003.docx]

Table S3. The information about the PEDV reference sequences.

| **Strain** | **GenBank No.** | **Location** | **Year** |
| --- | --- | --- | --- |
| SD-M | JX560761 | China | 2012 |
| HLJBY | KP403802.1 | China | 2015 |
| AH-M | KJ158152.1 | China | 2011 |
| DR13 | JQ023161 | Korea | 2009 |
| CHM2013-2 | KM887144.1 | China | 2013 |
| CV777 | AF353511 | Belgium | 1977 |
| LZC | EF185992 | China | 2006 |
| CH-S | JN547228 | China | 1986 |
| CH-SCMY-2018 | MH061343.1 | China | 2018 |
| CH-SCZY44-2017 | MH061338.1 | China | 2017 |
| GER-L03209-2019 | LR812926.1 | Germany | 2019 |
| KNU-1909 | MN844888.1 | South Korea | 2019 |
| FR-001-2014 | KR011756.1 | France | 2014 |
| PEDV 1842 | KY111278.1 | Italy | 2016 |
| 15V010-BEL-2015 | KR003452.1 | Belgium | 2015 |
| OKY-1-JPN-2014 | LC063847.1 | Japan | 2014 |
| USA-2014-IL-20697-P7 | KT591944.1 | United States | 2013 |
| KNU-1702 | MH052681.1 | South Korea | 2017 |
| OH851 | KJ399978.1 | United States | 2014 |
| USA-lowa106-2013 | KJ645695.1 | United States | 2013 |
| CH-EHuB4-2021 | PV107499.1 | China | 2021 |
| CH-JXYX-2024 | PV235414 | China | 2024 |
| CH-FS202201 | OR418363.1 | China | 2022 |
| CH-JY202201 | OR418364.1 | China | 2022 |
| CH hubei 2016 | KY928065.1 | China | 2016 |
| CH-HBTS-2017 | MH581489.1 | China | 2017 |
| CH-JXJA-2017 | MF375374.1 | China | 2017 |
| CH-ZMDZY-11 | KC196276.1 | China | 2011 |
| GDgh | MG983755.1 | China | 2018 |
| ZJ15XS0101-P1 | KX550281.1 | China | 2015 |
| SHXX1902 | MN841671.1 | China | 2019 |
| CHM2013 | KM887144.1 | China | 2013 |
| PEDV-LS | KM609211.1 | China | 2014 |
| USA-Indiana34-2013 | KJ645641.1 | United States | 2013 |
| NPL-PEDV-2013-P10 | KJ778616.1 | United States | 2013 |
| USA-Colorado-2013 | KF272920.1 | United States | 2013 |
| KNU-1305 | KJ662670.1 | South Korea | 2013 |
| USA-Ohio69-2013 | KJ645665.1 | United States | 2013 |
| PEDV-CHZ | KM609209.1 | China | 2013 |
| AH2012 | KC210145 | China | 2012 |
| BJ-2011-1 | JN825712.1 | China | 2011 |
| WHLL | MN037494.1 | China | 2017 |
| HUA-14PED96 | KT941120.1 | Korea | 2014 |
| YN1 | KT021227 | China | 2013 |
| YN90 | KT021231 | China | 2014 |
| AJ1102 | JX188454 | China | 2011 |
| PEDV-7C | KM609204 | China | 2011 |
| JS-A | MH748550 | China | 2017 |
| GD-1 | JX647847 | China | 2011 |
| GD-A | JX112709 | China | 2012 |
